# Transcriptome profiling in the spathe of *Anthurium andraeanum* ‘Albama’ and its anthocyanin-loss mutant ‘Xueyu’

**DOI:** 10.1038/sdata.2018.247

**Published:** 2018-11-13

**Authors:** Zhiying Li, Jiabin Wang, Yunliu Fu, Yu Gao, Hunzhen Lu, Li Xu

**Affiliations:** 1 Institute of Tropical Crop Genetic Resources, Chinese Academy of Tropical Agricultural Sciences, Danzhou 571737, Hainan, China; 2 Ministry of Agriculture Key Laboratory of Crop Gene Resources and Germplasm Enhancement in Southern China, Danzhou, 571737, Hainan, China; 3 Hainan Province Key Laboratory of Tropical Crops Germplasm Resources Genetic Improvement and Innovation, Danzhou 571737, Hainan, China; 4 Mid Tropical Crop Gene Bank of National Crop Resources, Danzhou, 571700, Hainan, China; 5 Nanjing Agricultural University, Nanjing 210095, Jiangsu, China

**Keywords:** RNA sequencing, Natural variation in plants

## Abstract

*Anthurium andraeanum* is a popular tropical ornamental plant. Its spathes are brilliantly coloured due to variable anthocyanin contents. To examine the mechanisms that control anthocyanin biosynthesis, we sequenced the spathe transcriptomes of ‘Albama’, a red-spathed cultivar of *A. andraeanum*, and ‘Xueyu’, its anthocyanin-loss mutant. Both long reads and short reads were sequenced. Long read sequencing produced 805,869 raw reads, resulting in 83,073 high-quality transcripts. Short read sequencing produced 347.79 M reads, and the subsequent assembly resulted in 111,674 unigenes. High-quality transcripts and unigenes were quantified using the short reads, and differential expression analysis was performed between ‘Albama’ and ‘Xueyu’. Obtaining high-quality, full-length transcripts enabled the detection of long transcript structures and transcript variants. These data provide a foundation to elucidate the mechanisms regulating the biosynthesis of anthocyanin in *A. andraeanum.*

## Background & Summary

*Anthurium andraeanum* is a popular cut flower and potted plant with a fantastic shape and impressive colours. It is a perennial and evergreen flower that originated in Columbia and Ecuador. The main attraction is its brilliantly coloured heart-shaped spathe and contrasting spadix. The common colours of *A. andraeanum* include red, pink, orange, white, brown and green. Elibox and Umaharan postulated that three dominant genes, R, O and M, controlled spathe colour. Furthermore, a white anthurium cultivar called ‘Acropolis’ suggested that white phenotypes resulted from regulatory rather than structural mutations^1,2^. A somaclonal variant called ‘Xueyu’ was generated during tissue culture of ‘Albama’; this mutant showed anthocyanin loss in the whole plant and a white spathe^3^.

Anthocyanins are widely found in the flowers, seeds, fruits and vegetative tissues of vascular plants. These soluble flavonoid pigments are responsible for red, blue and orange hues, and they can also participate in defence against a variety of biotic and abiotic stressors in plants. In *A. andraeanum*, the major colour pigments in the spathe are anthocyanins, particularly cyanidin and pelargonidin derivatives, of which the content and ratio determine the colour and its intensity^4^. The anthocyanin pathway has been extensively studied and is generally conserved over a wide range of plants. Generally, anthocyanin biosynthesis is regulated by the MYB-bHLH-WD40 (MBW) complex^5^. In addition, a complex regulatory network of positive and negative feedback mechanisms controlling anthocyanin synthesis in Arabidopsis has been described^6^. Furthermore, the transport and accumulation of anthocyanins affects the colour phenotypes of plants, but the mechanisms that control transport are unclear. Several anthocyanin pathway genes have been isolated in *A. andraeanum*. In our previous study, comparative transcriptome analysis was applied to determine the reason for anthocyanin loss in ‘Xueyu’. Moreover, transcriptome analysis was performed on a colour mutant of the anthurium cultivar ‘Sonate’^7^. Although transcriptome information was provided in our previous studies, the mechanisms regulating anthocyanin biosynthesis and spathe colour required further study.

We sequenced 4 cDNA libraries using the Pacific Biosciences RSII platform and 6 libraries using the Illumina HiSeq 4000 to characterize the spathe transcriptomes of ‘Albama’ and ‘Xueyu’ (Table 1). The long read sequencing produced 805,869 reads of insert, which were filtered to obtain 83,073 high-quality transcripts. The short read sequencing produced 347.79 M raw reads, and the results were assembled to yield 111,674 unigenes. The existing information regarding the *A. andraeanum* genome and transcriptome is limited, and thus, our data provided a valuable overview of additional transcriptome data from two cultivars of *A. andraeanum*. Moreover, our study identified transcripts differentially expressed between ‘Albama’ and ‘Xueyu’, which may be involved in the regulation of anthocyanin.

## Methods

The *A. andraeanum* plants were grown in the greenhouse of the Mid Tropical Crop Gene Bank of National Crop Resources located in Danzhou, China. The fully expanded spathes of the cultivars ‘Xueyu’ and ‘Albama’ were sampled. The sequencing work was performed by BGI Life Tech Co., Ltd (Shenzhen, China).

Total RNA extraction was performed using TRIzol (Promega, USA) and DNase I (Takara Bio, Japan). Using a Poly(A)PuristTM Kit (Ambion, now Life Technologies) and oligo-dT beads (Qiagen), the mRNA was isolated. Then the mRNA was fragmented and was used as a template to synthesize cDNA using a PrimeScript 1st Strand cDNA Synthesis Kit (Takara). The cDNA was purified and subjected to end preparation, single nucleotide adenine addition and adaptor ligation. After quality control with an Agilent 2100 Bioanalyzer and ABI StepOnePlus Real-Time PCR System, the library was sequenced using Illumina HiSeqTM 4000.

For SMRT Cell libraries construction, first-strand cDNA was synthesized using a SMARTer PCR cDNA Synthesis Kit (Clontech). Phusion High-Fidelity DNA Polymerase (NEB) was used to synthesize second-strand cDNA. The cDNA underwent BluePippin size selection (Sage Science) and then was normalized using the Trimmer-2 cDNA Normalization Kit (Evrogen) and amplified using large-scale PCR. Four fractions with normalized cDNA sizes of <1, 1-2, 2-3, and >3 kb were processed using the DNA Template Prep Kit (Pacific Biosciences of California, Inc.). After V2 primers and SA-DNA polymerase were linked to the templates, the complexes were then bound to magnetic beads for sequencing. Libraries with cDNA sizes <1 and >3 kb were sequenced with two cells, and the other libraries with one cell, using Pacific Bioscience RS II (Pacific Biosciences of California, Inc.).

The classification and filtering of long reads were performed using the SMRT analysis pipeline^8^. The raw long reads were filtered to reads of insert with minimum number of full passes (number of ends of SMRT Cell adapters were observed) of 0 and a minimum accuracy of 0.75. We then filtered the reads to cluster with a minimum length of 300 bp and a minimum phmmer score of 10 to detect the primer. The filtered reads were polished using the ICE algorithm, and the high-quality isoforms had a minimum Quiver^9^ accuracy of 0.99 for the libraries smaller than 3 kb and 0.98 for the libraries larger than 3 kb (Table 2). Then, cd-hit-est was used to remove the redundancy in the high-quality isoforms (Table 3).

For the short reads, we removed the noisy reads, which contained adaptors; more than 5% of unknown reads; and those in which the percentage of bases with a quality less than 15 was greater than 50% in a read using Trimmomatic^10^ (Table 4). Then, the reads were assembled into unigenes using Trinity^11^ (Table 5). Gene abundance was estimated by RSEM^12^ using the fragments per kb per million fragments (FPKM) method. Then, the differentially expressed genes were detected by NOISeq^13^ with a FDR ≤ 0.001 and fold change ≥ 2.

For functional annotation, the high-quality isoforms and unigenes were blasted against NT, NR, KEGG, COG and Swiss-Prot and subjected to InterProScan 5^14^. For the transcripts not mapped to any functional database, we predicted the CDS using ESTScan^15^ with Blast-predicted CDS as the model.

These methods above are expanded versions of descriptions in our related work^3,16^.

### Code availability

Trimmomatic: http://www.usadellab.org/cms/index.php?page=trimmomatic (version 0.38)

CD-HIT: http://www.bioinformatics.org/cd-hit/ (version 4.6.6)

Blast2GO: https://www.blast2go.com (version 2.5.0)

InterProScan: http://www.ebi.ac.uk/interpro (version 5.11)

Trinity: https://github.com/trinityrnaseq/trinityrnaseq (version 2.0.6)

## Data Records

The sequencing raw data of this study and our previous study^3^ were deposited in NCBI Sequence Read Archive (Data Citation 1). The project includes reads of insert from the long read sequencing and clean data from the short reads in FASTQ format, of which the four files with accession ID SAMN09296224, SAMN09296225, SAMN09296226 and SAMN09296227 are spathe transcriptome data from our previous study^3^. After removing of possible vector and NextGen sequencing primers contamination, 110,918 unigenes assembled from short reads were deposited in GenBank database (Data Citation 2). The transcript annotation data were deposited in figshare (Data Citation 3).

## Technical Validation

The total RNA used to construct the RNA-seq libraries was analysed, and samples with an RNA integrity number (RIN) more than 9 were used. The 347.79 M raw reads were filtered to 267.71 M clean reads, with a mean ratio of 77.1%. In addition, the short reads were de novo assembled to yield 384,791 unigenes in total; after removing redundancy, we obtained 111,674 unigenes.

**Figure 1:**
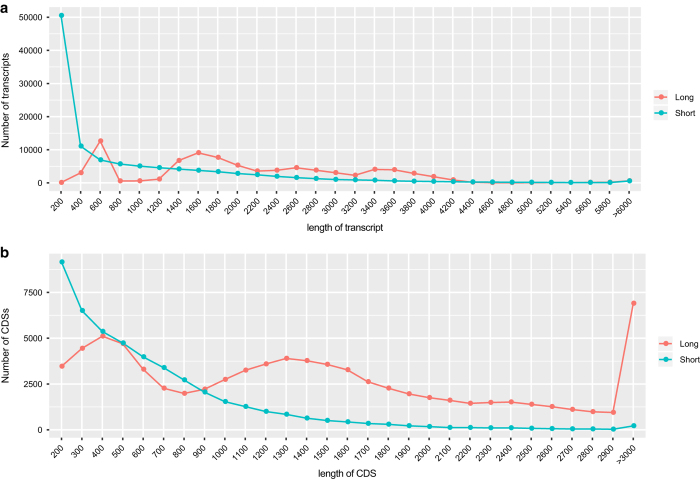
Length distributions of transcripts and CDS. (**a**) The transcript lengths of the HQ transcripts and unigenes. (**b**) The CDS length distribution of the transcripts.

**Figure 2:**
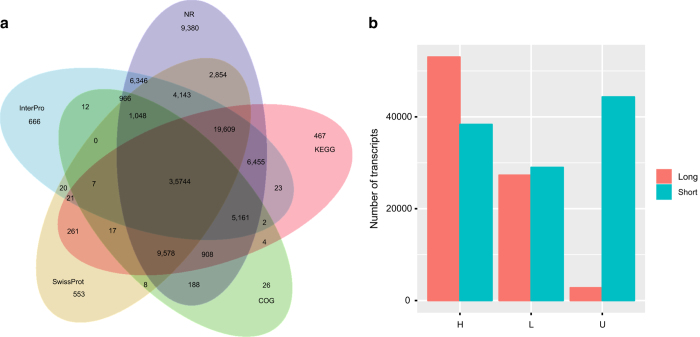
Annotation and Blast results for the HQ transcripts and unigenes. (**a**) HQ transcripts and unigenes were mapped to the NR, KEGG, COG, Swiss-Prot and InterPro databases. (**b**) HQ transcripts were mapped to unigenes with different similarity levels (H, identity more than 95%; L, identity less than 95%; U, no similarity).

Four long read libraries produced a total of 805,869 reads of insert, 387,845 full-length non-chimeric reads and 123,430 reads containing poly-A tails. All reads were clustered into 83,073 high-quality (HQ) transcripts. The length distributions of the HQ transcripts and unigenes are shown in Fig. 1a. The HQ transcripts were also mapped to the unigenes: 53,018 HQ transcripts and 38,348 unigenes shared high similarity (identity > 95%); 27,296 HQ transcripts and 28,991 unigenes showed low similarity; and 2,759 HQ transcripts and 44,335 unigenes had no similarity (Fig. 2b).

The transcripts, including HQ transcripts and unigenes, were mapped to the NR, KEGG, InterPro, COG and Swiss-Prot databases, and 35,744 transcripts could be mapped to all five databases (Fig. 2a). According to the annotations and predictions, 70,603 HQ transcripts and 55,031 de novo-assembled sequences were predicted to contain CDS; the distribution of CDS lengths is shown in Fig. 1b.

**Figure 3:**
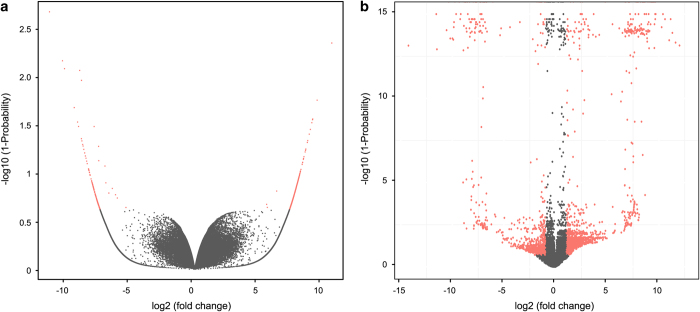
Volcano plot of differently expressed genes between ‘Xueyu’ and ‘Albama’. (**a**) The volcano plot of unigenes. (**b**) The volcano plot of HQ transcripts. The X-axis represents –log10-transformed significance. The Y-axis represents log2-transformed fold change. The red dot indicates differently expressed genes.

We performed differential expression analysis between samples of ‘Xueyu’ and ‘Albama’ of both HQ long reads and unigenes (Fig. 3). The differential expression analysis yielded 1,461 down- and 3,671 up- regulated HQ long reads and 199 down- and 435 upregulated unigenes. The expression and annotation information was deposited in figshare (Data Citation 3).

## Usage Notes

Because no reference genome is available for *A. andraeanum*, the raw long reads were corrected by clustering with the ICE algorithm. However, high-coverage short reads can also be used to correct errors in the long reads.

In our previous study, we compared the spathe transcriptome of stage 3 (flower protrudes from sheath) and stage 6 (the spathe is fully expanded) between ‘Xueyu’ and ‘Albama’ using Illumina short-read sequencing. To obtain high-quality, full-length transcripts, which enable the detection of long transcript structures and transcript variants, we performed isoform sequencing and Illumina short-read sequencing. The data of this study supplemented the transcripts and expression analysis data of the stage 6 spathe.

## Additional information

**How to cite this article**: Li, Z. *et al*. Transcriptome profiling in the spathe of *Anthurium andraeanum* ‘Albama’ and its anthocyanin-loss mutant ‘Xueyu’. *Sci. Data*. 5:180247 doi: 10.1038/sdata.2018.247 (2018).

**Publisher’s note**: Springer Nature remains neutral with regard to jurisdictional claims in published maps and institutional affiliations.

## Supplementary Material



## Figures and Tables

**Table 1:** Metadata of samples submitted to the NCBI Sequence Read Archive.

Source	Library strategy	Samples	Library layout	Platform	Instrument model	Biosample accession	Tissue
Albama	RNA-Seq	Albama_1	paired	ILLUMINA	Illumina HiSeq 4000	SAMN08322140	Spathe
Albama	RNA-Seq	Albama_2	paired	ILLUMINA	Illumina HiSeq 4000	SAMN08322141	Spathe
Albama	RNA-Seq	Albama_3	paired	ILLUMINA	Illumina HiSeq 4000	SAMN08322142	Spathe
Xueyu	RNA-Seq	Xueyu_1	paired	ILLUMINA	Illumina HiSeq 4000	SAMN08322143	Spathe
Xueyu	RNA-Seq	Xueyu_2	paired	ILLUMINA	Illumina HiSeq 4000	SAMN08322144	Spathe
Xueyu	RNA-Seq	Xueyu_3	paired	ILLUMINA	Illumina HiSeq 4000	SAMN08322145	Spathe
Albama and Xueyu	RNA-Seq	Mixed samples	single	PACBIO_SMRT	PacBio RS II	SAMN08322146	Spathe

**Table 2:** Summary of long read filtering.

Library	reads of insert	five prime reads	three prime reads	poly-A reads	full-length non-chimeric reads	full-length non-chimeric read length(bp)
between1k2k	258848	171,398(66.22%)	174,002(67.22%)	166,730(64.41%)	132,754(51.29%)	1836
between2k3k	172219	96,963(56.3%)	102,382(59.45%)	94,980(55.15%)	69,908(40.59%)	2967
between3k6k	174783	88,434(50.6%)	90,415(51.73%)	78,934(45.16%)	53,959(30.87%)	4026
under1k	200019	150,610(75.3%)	160,467(80.23%)	153,074(76.53%)	131,224(65.61%)	703

**Table 3:** Cluster of long reads.

Library	Cluster type	Total isoforms	Total base(bp)	Mean Quality	Mean isoform length(bp)	Mean Full length coverage
between1k2k	High quality	40898	74299859	0.9967	1817	2.8
between1k2k	Low quality	18000	38692106	0.3382	2150	1.01
between2k3k	High quality	20121	57171114	0.9953	2841	2.4
between2k3k	Low quality	21410	71870532	0.4915	3357	1.01
between3k6k	High quality	18403	68961773	0.9916	3747	1.81
between3k6k	Low quality	20589	93097977	0.4182	4522	1
under1k	High quality	17162	11707217	0.9991	682	5.1
under1k	Low quality	12006	9306751	0.3018	775	3.64

**Table 4:** Summary of short read filtering.

Sample	Total Raw Reads(Mb)	Total Clean Reads(Mb)	Total Clean Bases(Gb)	Clean Reads Q20(%)	Clean Reads Q30(%)	Clean Reads Ratio(%)
R1	52.25	44.24	6.64	98.61	95.75	84.66
R2	58.78	44.62	6.69	98.62	95.77	75.91
R3	58.78	44.13	6.62	98.59	95.68	75.08
W1	60.42	44.4	6.66	98.6	95.72	73.49
W2	58.78	45.22	6.78	98.48	95.39	76.93
W3	58.78	45.1	6.77	98.45	95.31	76.73

**Table 5:** Summary of short read de novo assembly.

Sample	Total Number	Total Length	Mean Length	N50	N70	N90	GC(%)
R1	61609	54847001	890	1561	939	329	48.36
R2	61048	55007752	901	1579	948	335	48.31
R3	60934	54374909	892	1560	939	330	48.35
W1	64474	57552118	892	1579	937	329	48.2
W2	68776	62144741	903	1620	964	330	47.49
W3	67950	61466947	904	1606	965	332	47.57
All-Unigene	111674	110235185	987	1875	1166	340	47.45
